# Being an adolescent and living with type 1 diabetes mellitus: A qualitative inquiry

**DOI:** 10.4102/curationis.v49i1.2852

**Published:** 2026-08-27

**Authors:** George J. Nkabinde-Thamae, Angie Ndhambi, Sipho W. Mkhize

**Affiliations:** 1 City of Ekurhuleni Metropolitan Municipality, Gauteng, South Africa; 2 School of Nursing and Public Health, Faculty of Health Sciences, University of KwaZulu-Natal, Durban, South Africa

**Keywords:** adolescents, type 1 diabetes mellitus, lived experiences, endocrine, primary healthcare facilities

## Abstract

**Background:**

Globally, adolescents experience peer pressure, drug and substance abuse and unemployment. In addition, other adolescents are living with chronic conditions such as type 1 diabetes mellitus (T1DM), which seems to have a negative impact on the quality of life they experience daily.

**Objectives:**

The study aimed to explore and describe the lived experiences of adolescents with T1DM.

**Method:**

This study employed a descriptive phenomenological approach. Unstructured individual interviews were conducted with 12 adolescents. The data were analysed using Giorgi’s data analysis steps.

**Results:**

Revealed shared struggles among adolescents living with T1DM. Their narratives illuminated the psychological, physical and social challenges they faced.

**Conclusion:**

This study unveiled the experiences of adolescents living with T1DM. The findings can help develop a more holistic clinical approach to managing adolescents with this condition, thereby enhancing their quality of life.

**Contribution:**

The study offered significant insights into the lives of adolescents living with T1DM and promotes awareness of their daily struggles, which gives healthcare organisations insight into what may be implemented to facilitate a supportive clinical environment that enhances the quality of life of adolescents living with type one diabetes mellitus.

## Introduction

Type 1 diabetes mellitus (T1DM) is an endocrine condition that mostly affects children and adolescents globally (Ward et al. 2022). This condition is explicitly characterised by the destruction of pancreatic beta cells, resulting in absolute insulin deficiency and persistent hyperglycaemia, requiring lifelong insulin therapy (International Diabetes Federation [IDF] 2025). The prevalence of adolescents living with T1DM as a chronic condition seems to be increasing among children and adolescents (Gomber et al. 2022). The 11th IDF highlighted that by 2025, around 9.5 million individuals (including children and adolescents) would be living with T1DM, an increase from 2021 (Ogle et al. 2025). The literature states there is a continuous yearly rise in the number of children and young people being diagnosed with T1DM (Gherbon 2025). Thus, Lan et al. (2026) argue that the high prevalence of T1DM underscores the need for healthcare organisations to prioritise effective management interventions for these individuals. Literature affirms that individual patient care is needed for adolescents living with T1DM, as their glucose levels constantly appear uncontrolled, putting these adolescents at greater risk of developing complications in the future (Ranasinghe et al. 2024).

Elhabashy et al. (2025) concur that the complications of diabetes mellitus have been noted among adolescents. Preventing complications related to hyperglycaemia and hypoglycaemia among adolescents requires adequate self-care, insulin administration and other healthy habits, such as eating healthily and getting sufficient rest, which should be promoted (American Association of Diabetes Educators 2019). Children and adolescents with T1DM experience numerous challenges, such as daily glucose monitoring, adherence to their treatment plan and adherence to an adequate diet plan, which often lead to anxiety, depression and stress among these adolescents (Akbarizadeh, Far & Ghaljaei 2022). Azar et al. (2024) observed that stress and anxiety affected the adherence plan of adolescents living with T1DM. Furthermore, adolescents living with diabetes mellitus have difficulty engaging with their peers and actively engaging in social life, which further promotes feelings of isolation and may lead to stress and even depression (Shattnawi & Mahassneh 2025). Harazneh, Malak and Ayed (2024) unveil that despite the challenges adolescents living with T1DM encounter, these adolescents must be seen and regarded as normal human beings and provided with support where needed. DeCosta, Grabowski and Skinner (2020) support the philosophy that adolescents living with T1DM should be regarded as complete and competent individuals whose identities are not defined by their illness. Despite the literature available on adolescents’ lived experiences with T1DM, the researchers could not find sufficient literature on adolescents living with T1DM, particularly in the Ekurhuleni Health District, Gauteng, South Africa. Because of the alarming prevalence and systemic challenges confronting this population, this research explored the experiences of adolescents within the City of Ekurhuleni, which provides demographic clarity, of the experiences of adolescents within this setting, as this researcher strictly adopts the adolescent definition established by the World Health Organization (WHO), who regard an adolescence an individual between the ages of 10 and 19 (Chauke et al. 2021; WHO 2014).

### Problem statement

The diagnosis of T1DM, among adolescents, is regarded as a demanding chronic condition, which often causes them physical and psychological discomfort as they attempt to navigate through the disease management (Kerimoğlu Yildiz, Ates Besirik & Azak 2026). In addition, a recent study affirms that managing T1DM in adolescents is challenging because of the physical, sexual and neurological development these individuals undergo (Bombaci et al. 2024). Unlike adults who have developed coping mechanisms for living with diabetes mellitus, adolescents often experience difficulties in living with and managing this chronic condition while navigating through developmental, physiological and emotional changes associated with adolescence (Hadad, Ali & Sayed 2021; Nyanful et al. 2026). This turbulence frequently exposes them to diabetes burnout, non-adherence to their treatment regimens and psychological discomfort, which negatively impacts their health outcomes (Azar et al. 2024; Orbea Cevallos et al. 2026). Despite the availability of clear guidelines and protocols globally for promoting metabolic control, the lived experiences of adolescents remain poorly understood and under-researched, especially within local healthcare settings (Le Roux et al. 2026; Morandi et al. 2024). Based on the literature above, without understanding the lived experiences of adolescents diagnosed with T1DM, there will be no contextual evidence to guide the development of supportive interventions these adolescents might need. The exploration of the subjective realities of these adolescents is therefore imperative to prevent potential health complications, identify and reduce psychological discomfort and empower adolescents to engage in effective, autonomous self-management.

### Theoretical framework

This study was guided by Dorothea Orem’s Self-Care Deficit Nursing Theory (2001), which was applied to explore and describe how adolescents experience living with T1DM as a chronic condition. The literature supports the relevance of Orem’s self-care deficit nursing theory for improving self-care practices among children and adolescents living with chronic conditions such as T1DM (Isik & Fredland 2023; Nkabinde-Thamae 2021; Nkabinde-Thamae & Downing 2024). This framework embraces three relevant constructs, such as **self-care** (adolescents’ daily management of T1DM, insulin administration, adherence to diet plan and blood glucose monitoring), **self-care deficit** (the developmental changes adolescents experienced, experiences of diabetes burnout, injection site reactions) and lastly **the nursing systems** (the support provided by nurses and doctors or even external such as family and friends). These constructs guided the study’s interview guide, providing the researchers with insight not only into the clinical aspects of this condition but also into adolescents’ daily self-care agency.

### Aim of the study

The study aimed to explore and describe the lived experiences of adolescents living with T1DM.

## Methodology

### Study design

The study was qualitative and utilised a phenomenological approach. The design is suitable because participants provide information-rich descriptions of living with diabetes as they experience and understand it daily. This approach assisted in exploring and describing insights into various occurrences while collecting participants’ experiences, perceptions and behaviours that are essential for the study at a particular moment (Polit & Beck 2020).

### The setting of the study

The study was conducted within the public healthcare sector of the Ekurhuleni Eastern Sub-District, located in the Gauteng Province of South Africa. Three tiers of care include health care institutions such as Primary Health Care (PHC) clinics, Community Health Centres (CHCs) and the Outpatient Departments (OPDs) of public hospitals. Data collection was limited to operational hours, specifically between 08:00 and 16:30. These settings were purposively selected because these public healthcare facilities are the primary points of care where adolescents access healthcare services, including the management of the chronic condition T1DM. Sampling across these three tiers provided a comprehensive view of adolescents’ lived experiences, capturing both local community care and specialised care within a hospital setting.

### Population and sampling strategy

A non-probability *purposive sampling method* was employed to select participants for this study. This approach was selected to recruit individuals with rich, firsthand lived experiences and the ability to provide valuable insights into living with T1DM, thereby addressing the study’s specific aims and objectives. To ensure rigorous sample selection, specific inclusion and exclusion criteria were established (Polgar & Thomas 2019). The detailed breakdown of these criteria is presented in Appendix 1, Table 1-A1.

### Data collection

The primary author conducted individual, face-to-face, semi-structured interviews from November 2023 to September 2024 to explore the lived experiences of adolescents living with T1DM. Data collection began only after receiving institutional ethical clearance, allowing the researchers to recruit relevant participants by distributing posters and flyers that included details of the study and the primary researcher’s contact information. These materials were hand-delivered by researchers to the designated healthcare institutions for display and distribution. The data collection process was facilitated by a central question, ‘*What are your experiences living with type 1 diabetes mellitus?*’, which allowed participants to freely share their experiences. In addition, the interview guide (Appendix 1, Table 2-A1) was employed to prompt discussions on key areas of interest, allowing the researchers to probe for deeper meaning in the phenomenon. To elicit deeper understanding, communication strategies such as probing, paraphrasing, active listening and summarising were used, allowing the researcher to clarify and explore nuances without leading the participants (Mabunda, Masondo & Mokoena-de Beer 2025; Nkabinde-Thamae & Downing 2026). The interviews were conducted in English and lasted for approximately 45–60 min. The interviews were conducted in a private room at the different institutions during scheduled appointment times or at an alternative location and time deemed convenient by the participant to ensure privacy and accessibility. Upon receiving participants’ consent, the interviews were audio-recorded, and participants’ nonverbal cues and body language were observed and documented in field observation notes, along with facial expressions, which are often lost in audio recordings. Two pilot interviews were conducted to pre-test the newly developed semi-structured interview guide and evaluate the researchers’ communication skills; however, no discrepancies were detected. The pilot data were excluded to prevent bias, maintain data consistency and uphold the rigour of the primary study. Data saturation, defined as the point at which no new information was elicited after the 10th interview, was confirmed by interviewing two additional participants (Nkabinde-Thamae & Downing 2025). The sample size of 12 participants was deemed appropriate, as it enabled a comprehensive exploration of participants’ experiences while maintaining depth and methodological rigour. This approach is consistent with established qualitative research principles, which prioritise depth, context and meaning over numerical representation. Although the study was conducted across clinics and hospitals that could yield a larger pool of participants, inclusion was guided by purposive sampling criteria to ensure that participants had direct, relevant experience with the phenomenon under investigation (adolescents living with T1DM).

### Data analysis

A professor of nursing with extensive experience in qualitative research analysed the data and met with the researchers to reach consensus on the identified themes. Giorgi’s descriptive thematic data analysis stages were employed (Giorgi, Giorgi & Morley 2017; Nene 2021). In stage 1, the researchers independently read all 12 transcripts to derive meaning from them. Thereafter (stage 2), the researchers grouped the 12 transcripts together using non-ambiguous language to indicate their intended meaning and perception. Subsequently, in stage 3, themes were generated using clear, descriptive words, which assisted the researchers (stage 4) in making sense of the data and in creating significant statements, thereby helping determine the themes relating to the experiences of adolescents living with T1DM.

### Ethical considerations

This study was approved by the Biomedical Research Ethics Committee of the University of KwaZulu-Natal (Reference number: BREC/00005790/2023). To access participants, the researchers formally requested approval from the Research Committee of the City of Ekurhuleni, and approval was granted by the Chair and Deputy Chair of the Committee (Reference number: 30/092023/01). The researchers furthermore had to register this study with the National Health Research Database (NHRD) for approval (Reference number: GP_202310_038). During the information session, the primary researchers explained the aims, objectives and potential risks of the study to participants. Despite the participants being of legal age, they were allowed to seek approval from their parents or any individual they regarded as a legal guardian. The primary researchers informed all participants that confidentiality, privacy, justice, anonymity and all other ethical principles would be upheld throughout the research process (Khuzwayo & Mkhize 2025; Mashazi et al. 2025; Tukisi 2024; Tukisi et al. 2025; Zondi & Mkhize 2025).

### Trustworthiness

In this study, the researchers promoted trustworthiness as follows: (1) *Credibility* was promoted through prolonged engagement, as data were collected from November 2023 until September 2024. Triangulation was achieved through multiple sources, including individual interviews, field notes and audio recordings. In addition, credibility was strengthened because the data analysis was conducted by a full professor in nursing with extensive knowledge and skills in qualitative research. Moreover, S.W.M. provided peer support and guidance, as he is a senior lecturer with a PhD and widespread experience in qualitative research. In addition, the researchers conducted member checking with the participants at an appropriate time, confirming telephonically whether the identified themes reflected their lived experiences of living with T1DM. (2) *Transferability* is apurposive sampling method, a clear description of the participants’ demographics and the research setting, as well as an in-depth description of the research methodology, promoted transferability of the study. (3) *Dependability* was enhanced by the clear and explicit discussion of the study results. (4) *Confirmability* was promoted by appropriate and relevant references. In addition, the researchers wrote reflexive notes that assisted them in reflecting on the participants’ experiences of living with T1DM. This helped them avoid bias, enhancing the objectivity of their findings by setting aside their own feelings, views and perceptions regarding the lived experiences of adolescents with T1DM.

## Results

Thematic analysis focused on the lived experiences of individuals (adolescents) living with T1DM revealed that adolescents struggle to live with T1DM. Their narratives illuminated the psychological, physical and social challenges of living with a chronic illness such as T1DM. A total of 12 adolescents participated in the study. This included 3 males, 8 females, and 1 participant who requested to withhold their gender information. Of note of concern, many of the participants were unemployed, which gives insight into the contributory attributes of their experiences of living with T1DM. Appendix 1, Table 3-A1 presents detailed information about the participants.

### Theme 1: Emotional and psychological burden

Participants described profound emotional challenges, stress and anxiety associated with living with T1DM. The verbatim quotes below give a clear lens into this theme.

‘Injecting myself feels like self-harm … I am ashamed to undress in front of anyone because of how my abdomen looks, which makes me sad.’ (Participant 1, 18 years, Male)

‘… what if it happens and no one is there to help; these are constant questions that come to my mind.’ (Participant 12, 18 years, Female)

‘I can handle everything, but stress associated with living with diabetes is not nice … I worry about keeping it controlled in order not to experience hypoglycemic attacks, but at times I feel so overwhelmed that I feel like committing suicide.’ (Participant 11, 18 years)

### Theme 2: Physical pain caused by routine needle injections

Participants in this study shared their daily routines for managing diabetes mellitus, which involved administering insulin as prescribed by a medical practitioner. However, they experienced challenges and some discomfort in administering the injections on time, and they felt the injections caused pain and discomfort or pain at the injection sites.

‘I would lie in bed, and my reminder on my phone goes off, that’s a reminder that I would need to inject myself. I understand why, but it’s so painful because I’ve been doing this forever.’ (Participant 5, 18 years, Female)

‘On a day-to-day basis, there is so much responsibility on my shoulders, but I think what is worse is the physical pain and scarring on my body; my fingers are not so sensitive when I take my glucose readings.’ (Participant 7, 18 years, Male)

‘My abdomen is covered in marks from injections … It’s not appealing. I somehow hate my body.’ (Participant 11, 18 years)

### Theme 3: Social self-isolation

The participants consciously isolated themselves from their peers, believing they were protecting themselves from possible negative attitudes (stigma and discrimination). In addition, participants reported that when with their peers, they were at risk of engaging in activities that could negatively affect their health.

‘I don’t think people who don’t live with diabetes, or any chronic conditions, would understand me, and my life in general, so I decided to spend time by myself and with close family members. Sometimes, I think the major reason I prefer being on my own is that adolescents not living with chronic conditions would force me to either drink alcohol, smoke, or even have the unhealthy food they have.’ (Participant 12, 18 years, Female)

Other participants added:

‘My friends find me boring because they would always say “live a bit normal,” which would mean drink alcohol, or smoke, so I am afraid of fitting in with their normal, because that would negatively affect my health.’ (Participant 3, 19 years, Female)

‘I don’t think my peers who are not living with diabetes can understand my daily struggles. We might be the same age, but our struggles are not. There is no reason for me to discuss my condition with them. They won’t be able to support me, but rather judge me and make my condition a joke, and that upsets me.’ (Participant 8, 19 years, Female)

### Theme 4: A desire for support to promote a holistic approach to care

The absence of emotional and practical support was a recurring theme, leaving participants feeling solely responsible for managing their condition. They yearned for a more profound support system from the healthcare system and their families. In addition, participants expressed the need for modernised interventions to test their glucose levels and administer insulin.

‘I am constantly reading online to empower myself … There is so much I need to figure out on my own. Healthcare organisations don’t even ask me about my hypoglycaemia attacks, and my parents, I think they’re tired because they’ve been taking care of me when I was younger.’ (Participant 9, 19 years, Female)

‘No one takes an interest in my condition … I feel like I am my own doctor or nurse. Sometimes it drains me physically and emotionally; support in terms of just reminding me about my clinic or doctor’s appointment would have been great, and despite the government seeing how many adolescents are negatively affected by diabetes, they could at least provide us with transportation, or a way to get medication to us, when we struggle to get to the clinic.’ (Participant 10, 19 years, Female)

‘Maybe a compulsory peer group will assist when one goes to collect treatment on a monthly basis.’ (Participant 11, 18 years)

## Discussion

This study intended to explore and describe the lived experiences of adolescents living with T1DM. Nsamba et al. (2022) stated that adolescents normally experience negative and positive feelings associated with living with T1DM. However, in this study, the participants seemed to have only negative experiences with living with T1DM. Most of the participants in this study expressed that living with T1DM affected their mental well-being to the extent that they constantly felt sad, depressed, anxious and, on some occasions, experienced suicidal thoughts. This phenomenon is supported by studies conducted in countries such as Turkey, Zambia and Kenya, which show that adolescents, in general, experience emotional and psychological stressors associated with living with T1DM (Palmer et al. 2022). In support of the participants’ reality, Malkawi, Bani Hani and Al Shikh (2025) affirm that adolescents living with T1DM experience severe depressive symptoms such as anxiety and depression.

The participants shared various reasons that contributed to their experiences of anxiety, sadness and depression because of living with T1DM. Adolescents with T1DM experienced emotional and psychological barriers as they found living with this chronic condition challenging, and they would constantly worry about possible future complications, such as amputations and dietary and alcohol restrictions (Anarte et al. 2020). Yani et al. (2025) further assert that adolescents face a negative self-image along with persistent fear and uncertainties regarding their future, resulting in emotional and psychological challenges. All the participants had lived most of their lives with T1DM but still could not cope with the routine of insulin injections. This correlates with the study conducted by Hanberger et al. (2021), who found that adolescents living with T1DM expressed that the routine insulin injections caused enormous bodily pain despite the site rotations. Adolescents reported that their nighttime insulin routine became more unbearable, as they often feared going into hypoglycaemia episodes if the insulin was not properly administered (Macaulay et al. 2020). Raja et al. (2020) report that despite the physical pain of injections that adolescents with type 1 diabetes experience, this routine contributes to their self-isolation, especially if they socialise with other adolescents who are not living with a chronic condition such as diabetes. Most of the participants expressed difficulty living with (T1DM), which resulted in them self-isolating from others despite the negative impact associated with social isolation.

Literature reveals that adolescents living with T1DM often experience loneliness and sadness because of social isolation (Geukens et al. 2022). Furthermore, Bonell et al. (2019) found that, because of social isolation, adolescents with T1DM constantly yearned for a sense of belonging, especially from peers. In this study, despite participants’ desire to fit in with their peers, they still felt the need to isolate themselves, as they felt misunderstood and different. Their peers could not relate to their daily challenges and the routine of living with and managing them (Holmström & Söderberg 2021). Despite the negative impact of social isolation, it can be beneficial to adolescents living with T1DM, as studies have found that when socialising with their peers, adolescents living with T1DM often engage in unhealthy activities such as drinking alcohol, eating the wrong foods and forgetting to take their insulin, which frequently leads to hypoglycaemia or hyperglycaemia episodes (Hanna & Hansen 2020). Strand, Broström and Haugstvedt (2019) disputed the findings that revealed the negative impact of peers on adolescents living with T1DM. They found that adolescents should not isolate themselves from their peers because strong support from friends and others can help adolescents living with T1DM gain valuable support. Similar studies concur that it is imperative that adolescents living with T1DM engage with others, especially peers, to foster a sense of belonging and support (Hagger et al. 2022; Spitz, Winkler Metzke & Steinhausen 2020).

The participants explicitly shared their lived experience with living with T1DM, which included the perception that they were not supported by their peers, family members and even healthcare professionals. The researchers found this somewhat contradictory, as the participants reported self-isolating from others, especially their peers, which could exacerbate the possible lack of support. Additionally, the participants felt that other interventions and support were necessary to help them live effectively with T1DM. Despite the various interventions needed to promote their quality of life, interventions that would promote their emotional and psychological well-being appeared to be a priority, as many adolescents living with T1DM appeared to have experienced feelings such as sadness, frustration and anxiety because of living with T1DM (Dimitri et al. 2020). In support of these findings, Dimitri et al. (2020) inferred that psychological support is imperative as it will not only assist adolescents in coping with stress, anxiety and depression but also guide these adolescents on how to cope with possible future complications associated with T1DM. Ali (2023) noted that, as a sign of support, healthcare workers, especially professional nurses, should adopt a telenursing approach in which nurses engage with adolescents living with T1DM weekly to assess their well-being and determine what assistance they may need. Additionally, healthcare organisations need to create continuous health education to empower these adolescents regarding correct diet, alcohol intake and the importance of physical activity to prevent possible complications such as hypoglycaemia (Montt-Blanchard et al. 2022). In addition to the desired support adolescents appeared to need, they needed to engage with their peers rather than isolate themselves.

A longitudinal study found that healthcare organisations must develop sustainable, focused support groups to promote interaction among adolescents living with T1DM, enabling them to share their daily challenges and to receive individual interventions to overcome them (Luo et al. 2024). In this study, the adolescents further shared with the researchers’ other aspects where they needed assistance or support, as they sometimes struggled to honour their monthly appointments because of transportation issues getting to and from the healthcare institution. Transportation continues to be a significant obstacle to continuity of care, as adolescents living with T1DM are not always easily accessible to a healthcare organisation for treatment collection, which puts them at risk of going without medication. In addition, in many African countries, including South Africa, it has been noted that many public health organisations still use the traditional manner of monitoring glucose or insulin administration rather than advanced interventions that do not include invasive procedures such as pricking and intramuscular administration of insulin (Castellanos et al. 2020). Hanberger et al. (2021) affirmed that the availability of advanced digital interventions would assist adolescents living with T1DM in effectively managing their chronic condition, with minimal supervision or assistance. The researchers cited demographic information showing that most participants were unemployed, concluding that most of the desired support needed to achieve a quality of life depends on a healthcare institution prioritising T1DM among adolescents.

### Strengths and limitations of the study

This study provided insight into the lived experiences of adolescents living with T1DM. It revealed the daily challenges they encountered, which directly impacted their quality of life. The findings of this study may support better clinical management of adolescents with T1DM. This study was conducted solely through in-depth individual interviews with 12 adolescents living with T1DM, as data from the pilot interviews (two participants) were excluded from the analysis. Adolescents living with T1DM who accessed private hospitals or health facilities could have benefited from a larger sample, which may have contributed to this phenomenon. In addition, the researchers did not have a plan to follow up with participants who were referred for counselling to maintain continuity of care.

## Recommendations

### Further research

The results that unveil the emotional impact the chronic condition T1DM had on these participants indicate a need for in-depth research to be done on the emotional well-being of adolescents living with T1DM. In addition, research such as recommendations to improve the quality of life and experience of adolescents living with T1DM can be undertaken.

### Policy

The findings of this study revealed that adolescents living with T1DM struggled to adapt to their daily routines. Theme 1 specifically revealed the emotional and psychological struggles the participants experienced because of living with T1DM. Policies at the national and provincial levels need to be extensively developed and implemented to cover the psychological well-being of adolescents living with T1DM and to include guidance on how professional nurses and other healthcare workers should screen and assess adolescents’ mental well-being during subsequent visits.

### Practice

Considering the findings of the study, it appears that adolescents living with T1DM find it extremely difficult to live with this chronic condition. Healthcare institutions, including primary healthcare facilities, should be more sensitive to these adolescents when they visit.

## Conclusion

This study represents the initial exploration and description of adolescents living with T1DM. The findings indicated that adolescents struggled significantly with living with this chronic condition. The findings furthermore underscore the importance of healthcare institutions acknowledging and understanding the experiences of adolescents living with this chronic condition. Supportive interventions need to be developed and implemented to promote and sustain the quality of life of adolescents living with chronic conditions and necessarily also for adolescents living with T1DM.

The views and opinions expressed in this article are those of the authors and are the product of professional research. The article does not necessarily reflect the official policy or position of any affiliated institution, funder, agency or the publisher. The authors are responsible for this article’s results, findings and content.

## Appendix 1

**TABLE 1-A1:** Eligibility criteria.

Criterion category	Inclusion criteria	Exclusion criteria
Age	• Late adolescents aged 18–19 years at the time of the interview.	• Individuals under 18 years or older than 19 years.
Clinical diagnosis and treatment	• Confirmed diagnosis of T1DM. • Prescribed a routine daily insulin regimen.	• Individuals diagnosed with Type 2 diabetes. • Individuals on oral hypoglycaemic agents only.
Duration of illness	• Diagnosed with T1DM for 12 months or longer to ensure a holistic reflection of lived experiences.	• Diagnosed with T1DM for less than 12 months (early adjustment phase).
Sector of care	• Dependent on public healthcare institutions (hospitals, outpatient departments, CHC or primary clinics).	• Utilising private healthcare institutions for primary diabetes care.
Participation and consent	• Attended the planned study information session. • Provided independent, written informed consent.	• Declined to attend the information session. • Refused to provide informed consent.

CHC, community health centre; T1DM, type 1 diabetes mellitus.

**TABLE 2-A1:** Interview guide.

Number	Example of interview questions
1	What are your experiences living with type 1 diabetes mellitus?
2	What comes to mind when you think about living with type 1 diabetes mellitus?
3	What do you mean, you said you have no support?
4	How would support assist you to live better with type 1 diabetes mellitus?
5	How does self-isolation help you?
6	What would have happened if you engaged with your peers?
7	Have you communicated your challenges to them?
8	What brings on the fear of you going into a hypoglycaemia state?
9	I heard mixed feelings when you mentioned insulin injections; what emotions do you experience most of the time when you are injecting yourself?
10	How would modernise interventions assist in keeping your glucose levels under control?

**TABLE 3-A1:** Detailed information about the participants.

Participant	Age	Gender	Estimate the number of years living with type 1 diabetes mellitus	Employment status
Participant 1	18	Male	± 17 years	Employed (Part-time)
Participant 2	19	Female	± 12 years	Unemployed
Participant 3	19	Female	More than 2 years, but not sure of the specific years	Employed
Participant 4	18	Female	± 15 years	Employed
Participant 5	18	Female	± 12 years	Employed (Part-time)
Participant 6	18	Male	± 15 years	Employed (Part-time)
Participant 7	18	Male	± 14 years	Unemployed
Participant 8	19	Female	More than 4 years, but not sure of the specific years	Unemployed
Participant 9	19	Female	± 17 years	Unemployed
Participant 10	19	Female	± 10 years	Employed
Participant 11	18	Withheld	± 3 years	Employed (Part-time)
Participant 12	18	Female	More than 6 years, but not sure of the specific years	Unemployed
